# Author Correction: Temperature extremes of 2022 reduced carbon uptake by forests in Europe

**DOI:** 10.1038/s41467-023-42798-y

**Published:** 2023-11-01

**Authors:** Auke M. van der Woude, Wouter Peters, Emilie Joetzjer, Sébastien Lafont, Gerbrand Koren, Philippe Ciais, Michel Ramonet, Yidi Xu, Ana Bastos, Santiago Botía, Stephen Sitch, Remco de Kok, Tobias Kneuer, Dagmar Kubistin, Adrien Jacotot, Benjamin Loubet, Pedro-Henrique Herig-Coimbra, Denis Loustau, Ingrid T. Luijkx

**Affiliations:** 1 University of Groningen, Centre for Isotope Research, Groningen, 8481 NG The Netherlands; 2 Wageningen University, Meteorology & Air Quality Dept, Wageningen, 6700 AA The Netherlands; 3 Université de Lorraine, AgroParisTech, INRAE, UMR Silva, 54000 Nancy, France; 4 Functional Ecology and Environmental Physics, Ephyse, INRA, Villenave d’Ornon, France; 5 Copernicus Institute of Sustainable Development, Utrecht University, Utrecht, The Netherlands; 6 UMR CEA-CNRS-UVSQ, Laboratoire des Sciences du Climat et de l’Environnement, Gif sur Yvette, France; 7 Max Planck Institute for Biogeochemistry, Jena, Germany; 8 Faculty of Environment, Science and Economy, University of Exeter, Exeter, UK; 9 ICOS ERIC, Carbon Portal, Geocentrum II, Sölvegatan 12, SE-22362 Lund, Sweden; 10 Deutscher Wetterdienst, Hohenpeissenberg Meteorological Observatory, Hohenpeissenberg, Germany; 11 Sol, Agro et hydrosystèmes, Spatialisation (SAS), UMR 1069, INRAE, Institut Agro, Rennes, France; 12 Université Paris Saclay, AgroParisTech, INRAE, UMR 1402 ECOSYS, 91120 Palaiseau, France; 13 ISPA, Bordeaux Sciences Agro, INRAE, F-33140 Villenave d’Ornon, France

**Keywords:** Carbon cycle, Atmospheric science

Correction to: *Nature Communications* 10.1038/s41467-023-41851-0, published online 06 October 2023

In this article the grant number 4000140982/23/I-EF relating to the ESA Carbon-RO for Ana Bastos, Philippe Ciais, and Stephen Sitch was omitted. The original article has been corrected.

Additionally, Auke M. van der Woude and Wouter Peters should have been denoted as equally contributing authors. The original article has been corrected.

