# Supplementary material for: Temperature extremes of 2022 reduced carbon uptake by forests in Europe
Source: Nat Commun. 2023 Oct 6;14:6218. doi: 10.1038/s41467-023-41851-0 (PMC10558467; doi:10.1038/s41467-023-41851-0)
Supplement: Supplementary file 1 — Supplementary information [file 41467_2023_41851_MOESM1_ESM.pdf]

Supplementary Materials for: Temperature extremes of 2022 reduced carbon uptake by forests in Europe

Auke M. van der Woude[1,2,+], Wouter Peters[2,1,+,*], Emilie Joetzjer[3], Sébastien Lafont[4], Gerbrand Koren[5], Philippe Ciais[6], Michel Ramonet[6], Yidi Xu[6], Ana Bastos[7], Santiago Botía[7], Stephen Sitch[8], Remco de Kok[2,9], Tobias Kneuer[10], Dagmar Kubistin[10], Adrien Jacotot[11], Benjamin Loubet[12], Pedro-Henrique Herig-Coimbra[12], Denis Loustau[13], Ingrid T. Luijkx[2]

[1]University of Groningen, Centre for Isotope Research, Groningen, 8481 NG, The Netherlands,
[2]Wageningen University, Meteorology & Air Quality Dept, Wageningen, 6700 AA, The Netherlands,
[3]Université de Lorraine, AgroParisTech, INRAE, UMR Silva, 54000 Nancy, France,
[4]Functional Ecology and Environmental Physics, Ephyse, INRA, Villenave d'Ornon, France ,
[5]Copernicus Institute of Sustainable Development, Utrecht University, Utrecht, The Netherlands,
[6]UMR CEA-CNRS-UVSQ, Laboratoire des Sciences du Climat et de l'Environnement, Gif sur Yvette, France,
[7]Max Planck Institute for Biogeochemistry, Jena, Germany,
[8]Faculty of Environment, Science and Economy, University of Exeter, Exeter, UK,
[9]ICOS ERIC, Carbon Portal, Geocentrum II, Sölvegatan 12, SE-22362 Lund, Sweden,
[10]Deutscher Wetterdienst, Hohenpeissenberg Meteorological Observatory, Hohenpeissenberg, Germany ,
[11]Sol, Agro et hydrosystèmes, Spatialisation (SAS), UMR 1069, INRAE, Institut Agro, Rennes, France,
[12]Université Paris Saclay, AgroParisTech, INRAE, UMR 1402 ECOSYS, 91120 Palaiseau, France,
[13]ISPA, Bordeaux Sciences Agro, INRAE, F-33140, Villenave d'Ornon, France,

[*]corresponding author: Wouter.Peters@wur.nl
[+]these authors contributed equally to this work

A Selection of regions

Table S1. Area (million square kilometre), Biomass (GtC) and SPEI for the selected areas. Biomass is taken from Avitabile et al. (2014)[1]. For the Centre region, SPEI is given as 2018/2022

Region	North	Centre	South	East
Area	1.9	0.83	1.3	0.84
Biomass	5.1	1.9	2.2	3.1
SPEI	-2.2	-2.1/-1.9	-2.2	-1.8

B Soil moisture and VPD anomalies

Europe experienced lower than normal soil moisture in 2022 from January onwards in the South, and over the Center and East from March onward. This is indicated by the anomalies shown in Figure S1. However, the soil moisture anomalies did not develop as strongly as in 2018 in Summer, as shown in Figure S2. Whereas JJA of 2018 shows soil moisture anomalies outside the 2σ range in the drought-affected area, soil moisture anomalies outside the 2σ range are shown in 2022 in a smaller area (Fig S2). We note that observations from the SMAP L-band satellite[2] show very similar patterns, with an area-averaged correlation of 0.98, 0.97 and 0.92 for the South, East and North regions, respectively.

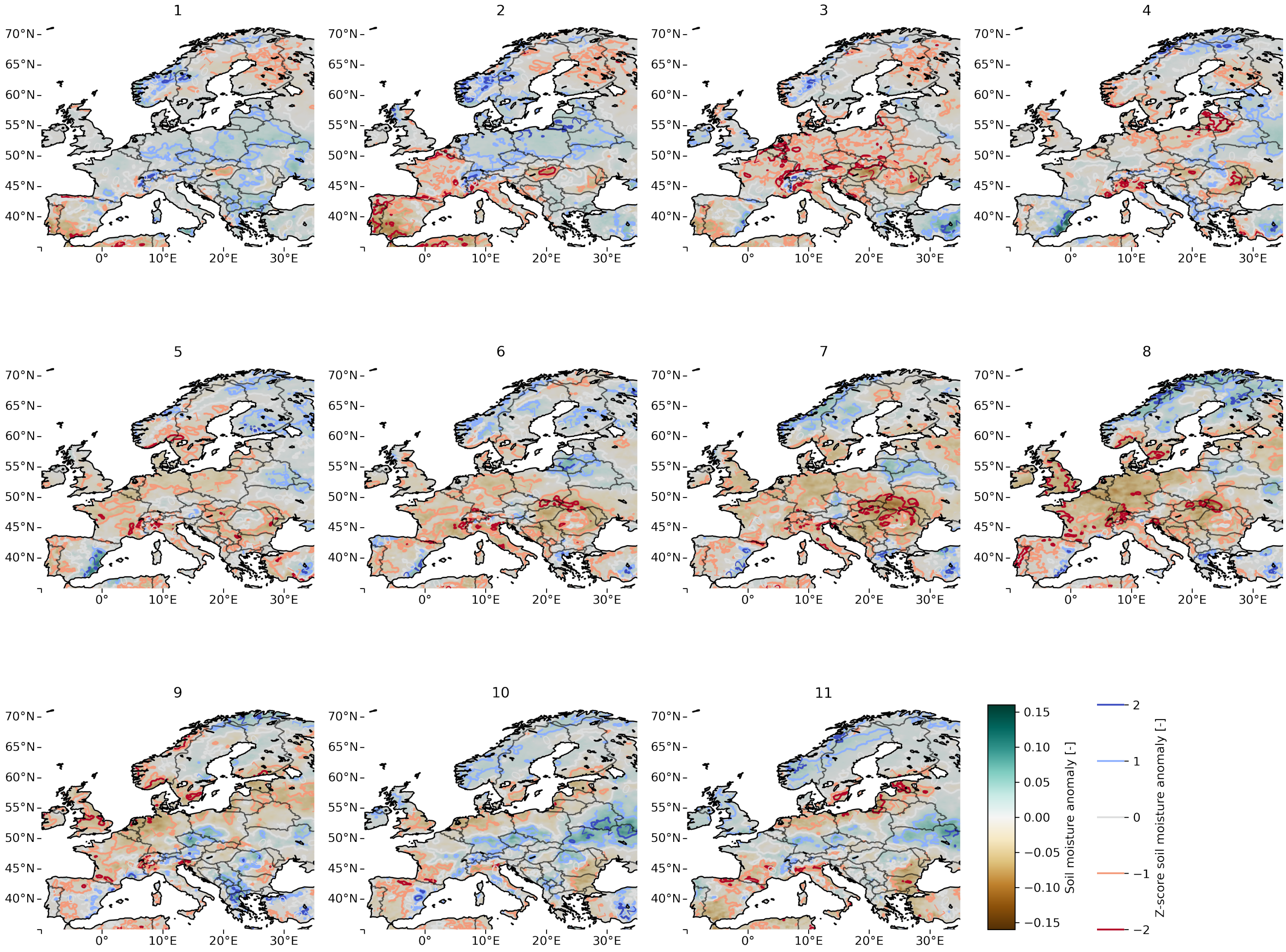

Figure S1. ERA5-Land[3] monthly detrended soil moisture anomalies for January-November 2022 (1-11, respectively), relative to 2000-2021 over the first meter of soil. The contours indicate standard deviations from -2σ to 2σ. The month number is shown in the subtitle of the plot.

Contrary to soil moisture, vapor pressure deficit, a measure of atmospheric drought, was much stronger in 2022 than in 2018. In JJA 2022, the VPD was highest over all years considered in 45 / 51 / 15% of the Centre / South / East regions. We calculate VPD from the ERA5 reanalysis data[4]. In JJA 2018, VPD was much less strong, being the highest in only 36 / 24% of the regions North and Centre, respectively. VPD anomalies larger than 1σ were ubiquitous over the European continent in JJA 2022, and VPD anomalies larger than 2σ were found over the Centre, South and Eastern regions (Figure S3).

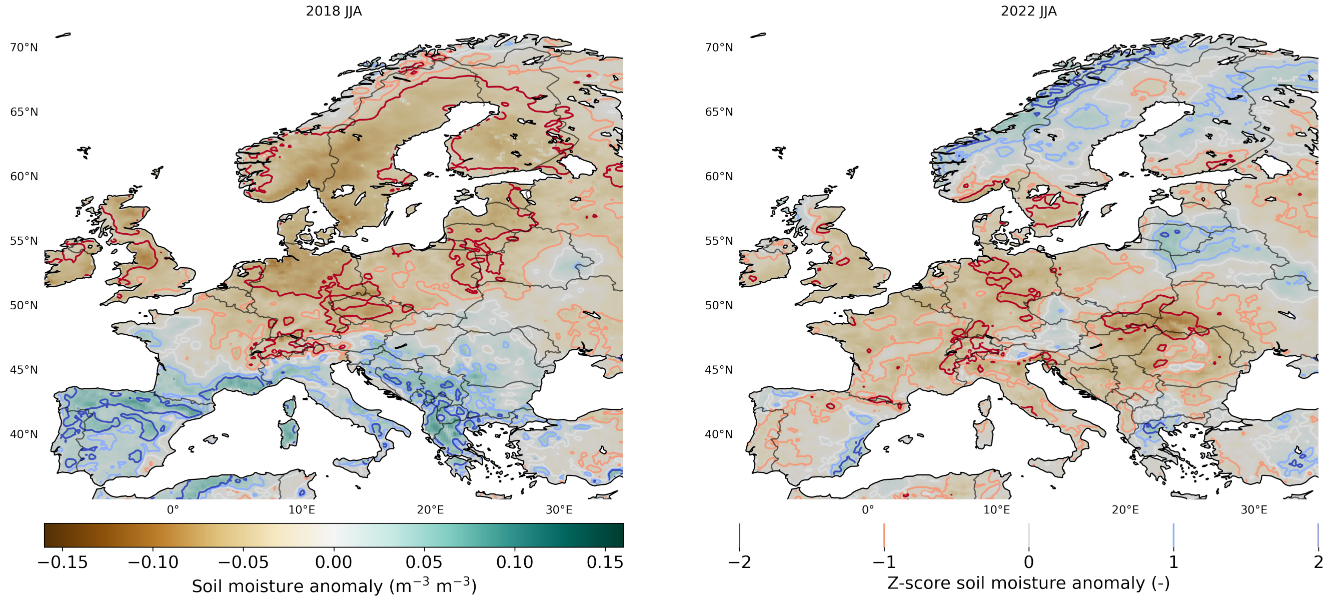

Figure S2. ERA5-Land soil moisture anomalies for JJA 2018 (left) and 2022 (right), relative to 2000-2022 (not including 2018 and 2022). Soil moisture is calculated over the first meter of soil. The contour lines indicate standard deviations from -2σ to 2σ.

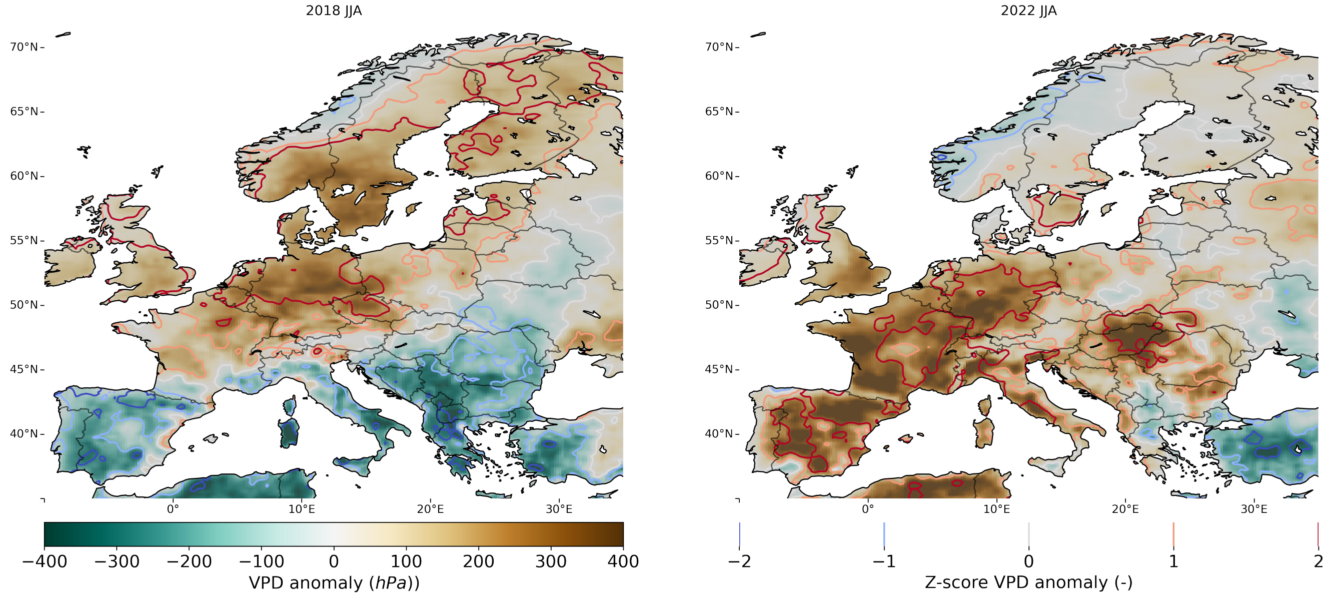

Figure S3. VPD anomalies from ERA-5 for JJA 2018 and 2022, relative to 2000-2022 (not including 2018 and 2022). Linear trends in the data are removed before calculating the anomaly. The red, pink, white, light blue and blue contours indicate anomalies outside the [-2, -1, 0, 1, 2] σ range, respectively

C Atmospheric measurements

id	lat	lon	altitude	class	name
LUT	53.4036	6.3528	21.0	C	Lutjewad the Netherlands
MHD	53.3261	-9.9036	20.0	S	MaceHead Ireland
GAT	53.0657	11.4429	401.0	T	Gartow Germany
HPB	47.8011	11.0246	1236.0	T	Hohenpeissenberg Germany
HTM	56.0969	13.4189	254.0	T	Hyltemossa Sweden
SMR	61.8474	24.2947	208.0	T	Hyytiala Finland
KRE	49.5720	15.0800	784.0	T	Kresin Czech Republic
LIN	52.1663	14.1226	162.0	T	Lindenberg Germany
NOR	60.0864	17.4794	146.0	T	Norunda Sweden
OPE	48.5619	5.5036	510.0	T	OPE ANDRA France
PUI	62.9096	27.6549	316.0	T	Puijo Finland
TRN	47.9647	2.1125	211.0	T	Trainou France
BIS	44.3781	-1.2311	120.0	C	Biscarrose France
FKL	35.3378	25.6694	250.0	S	Finokalia Greece
SAC	48.7227	2.1420	260.0	T	Saclay France
UTO	59.7839	21.3672	65.0	S	Uto Baltic Sea Finland
IPR	45.8147	8.6360	310.0	T	Ispra Italy
OHP	43.9310	5.7120	750.0	T	Obervatoire de Haute Provence France
CMN	44.1936	10.6999	2825.0	M	Monte Cimone Italy
JFJ	46.5475	7.9851	4290.0	M	Jungfraujoch Switzerland
PUY	45.7719	2.9658	1865.0	M	Puy de Dome France
KIT	49.0915	8.4249	310.0	T	Karlsruhe Germany
OXK	50.0200	11.8083	1185.0	M	Ochsenkopf Germany
STE	53.0431	8.4588	281.0	T	Steinkimmen Germany
TOH	51.8088	10.5350	948.0	T	Torfhaus Germany
JUE	50.9102	6.4096	218.0	T	Juelich Germany
CRA *	43.1298	0.3692	600	T	Centre de Recherches Atmosphériques France

Table S2. ICOS atmospheric sites used in this study, including their latitude, longitude, station class (M for mountain, T for tall-tower, UP urban/polluted stations, C for coastal sites, S for ocean) and full name of the station[5]. Sites indicated with a * are not included in the CO_2 analyses for a lack of STILT footprints.

Beside CO_2 also carbon monoxide, CO, was measured at some stations. CO is a tracer for incomplete burning, e.g. due to wildfire activity. The large wildfires in France, as described in Section 'Fires', are seen as enhanced CO mole fractions (Figure S4).

C.1 Calculation of background influence

Measured anomalies in atmospheric CO_2 mole fractions are comprised of anomalies in fluxes and in background CO_2 levels, which can either be anomalous themselves or result from anomalous transport patterns in the atmosphere. To account for this, we subtract the anomaly in background CO_2 mole fractions from the observed CO_2 mole fraction anomaly.

Background influences were calculated by transporting a climatological background with the Lagrangian particle dispersion model STILT[6], driven by the integrated forecast system (IFS, following the IFS cycle development; for more information, see https://www.ecmwf.int/en/publications/ifs-documentation, last access: 15 March 2023), as described in van der Woude et al (2022, referred to as vdW2022 from hereon)[7]. The climatological background was taken from the optimised CO_2 mole fractions from the CarbonTracker Europe (CTE2022) contribution to GCP2022[8]. This climatological (2019-2021) background represents gradients in atmospheric CO_2 mole fractions in the north-south, and east-west directions. As we subtract the linear trend in atmospheric growth rate, the absolute magnitude of the background is removed, and mainly latitudinal differences, combined with differences in wind direction, drive the importance of the background. Table S3 shows that the background is of limited importance and is generally smaller than 0.5 ppm.

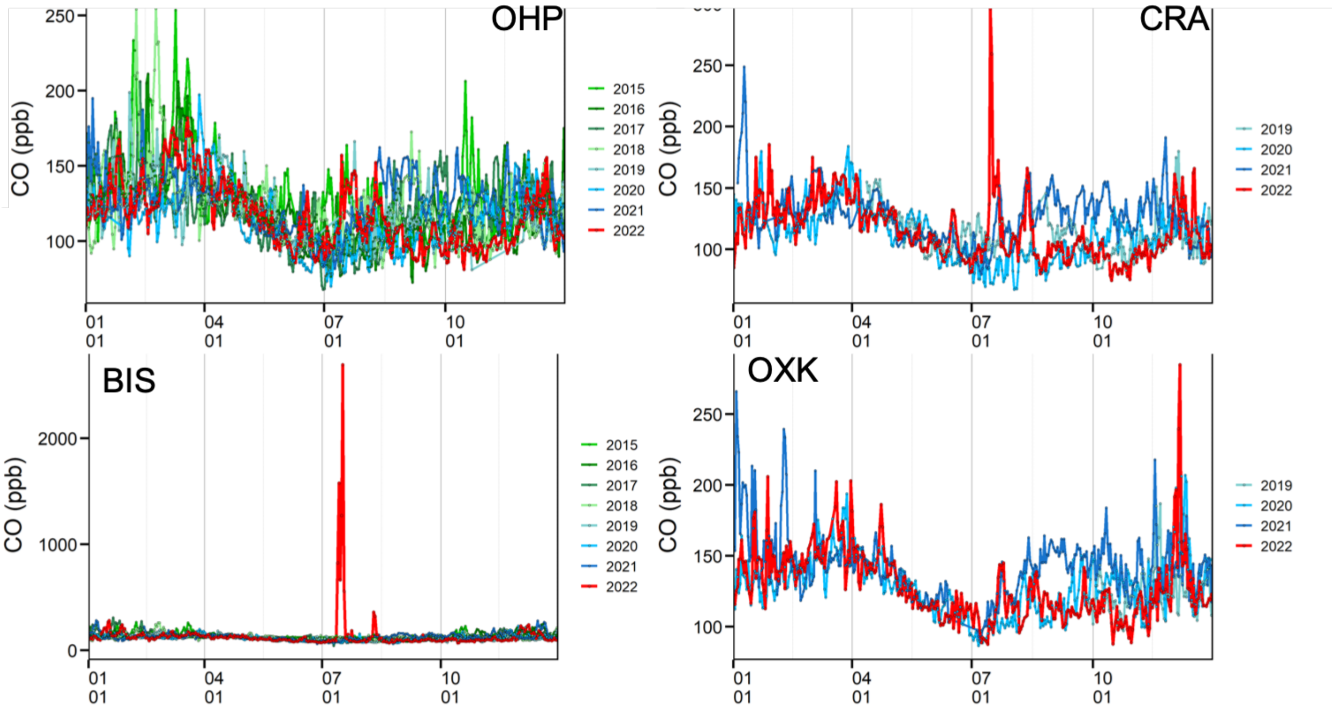

Figure S4. 1-day averaged CO mole fractions measured at four sites (OHP: Obervatoire de Haute Provence, France; CRA: Centre de Recherches Atmosphériques, France; BIS: Biscarrose, France; OXK: Ochsenkopf, Germany) for multiple years (colours). The x-axis shows the month of the year and the day of the month. The sites are listed in Table S2.

Table S3. Relative and absolute importance of the background influence on observed atmospheric CO_2 mole fraction anomalies for used atmospheric stations for selected months in 2022. The range indicates the 5-95 percent quantiles of influence, expressed as percentage of the observed anomaly due to background CO_2 anomalies. The Absolute influence indicates the median of the background influence of all stations, expressed as ppm CO_2. The mean influence indicates the mean influence of the background relative to transported flux anomalies. The data uses only representative hours over all used stations. The stations are listed in Table S2.

Month	Relative influence (%)	Absolute influence (ppm)	Mean influence (%)
6	1.22 - 82.53	-0.33	23.77
7	0.94 - 66.54	-0.06	18.82
8	0.73 - 81.12	0.02	21.03
9	1.37 - 86.71	0.34	24.06
10	1.43 - 96.05	-0.62	27.33
11	0.68 - 118.4	-0.31	28.13

Table S4. Similar to S9, but for the high-resolution SiB4, selected at the gridcells the stations are in.

	P ratio		Rg W m^{-2}		Tair K	
	JJA	SON	JJA	SON	JJA	SON
station name						
BE-Vie	-0.60	0.37	10.48	-4.77	1.74	1.47
FR-FBn	1.21	0.14	-4.71	-10.09	2.17	1.26
BE-Bra	-0.59	-	21.38	-	0.38	-
IT-SR2	-0.25	-0.12	8.36	41.49	2.18	1.56
FR-Fon	-0.20	-0.05	12.11	5.59	2.31	2.53
FR-Bil	-0.49	-0.25	20.52	39.84	3.74	2.75
FI-Hyy	-0.05	-0.08	5.70	0.81	1.22	0.61
DE-Tha	-0.26	0.38	3.12	31.81	1.00	1.04
FR-Hes	-0.22	0.22	8.70	14.56	1.97	3.09
DE-HoH	-0.46	0.66	11.96	10.29	1.24	1.27
FR-Pue	0.14	-0.23	-2.57	-15.53	2.54	1.80
CH-Dav	-0.40	0.08	25.20	-5.75	1.61	2.22
SE-Htm	-0.33	-0.00	12.14	7.72	0.88	1.40
DK-Sor	-0.11	-0.16	-1.06	8.09	0.51	1.41

D SiB4 biosphere model

We use a spatially downscaled version of the SiB4 biosphere model[9], as described in vdW2022. The resulting product has a spatial resolution of 0.1 by 0.2 ° and hourly output. Additional to the NEE, as used in vdW2022, we also analyse GPP, TER, soil moisture stress and leaf relative humidity (VPD) stress, as well as the driver data for SiB4 (ERA5 reanalysis data[4]). Note that SiB4 outputs the net ecosystem productivity (NEP = GPP - TER), whereas EC towers measure the net ecosystem exchange (NEE, which includes fires). As the influence of fires is generally small on EC sites, they can be compared directly and in this work we refer to SiB4 fluxes as NEE to remain consistent with SM2020. We note NEE from the atmospheric perspective, i.e. uptake by plants is negative and positive NEE indicates emissions into the atmosphere.

D.1 Model performance at larger scales

SiB4 shows a good drought response for the 2018 drought, which is demonstrated in SM2020 and vdW2022. Both studies show that transported SiB4 NEE fluxes are in good agreement with tower measurements of CO_2 mole fractions. There is a similar good agreement between the NEE anomalies produced by SiB4 and those from the simple inversions performed in this paper (Section E), indicating that the large-scale absolute response of the biosphere is well represented in SiB4, also during droughts. Furthermore, the spatial patterns of GPP anomalies agree well with those of NIRv for 2022 (see Figures 4 and S7) and with that of SIF for 2018[10].

The spatial correlation between GPP anomalies as calculated by SiB4 and those of NIRv is 0.78 (N=41, P=10^{-9}). We calculated the correlation by first calculating GPP and NIRv anomalies for JJA with respect to 2016-2021. We averaged the anomalies temporally and removed gridcells at which SiB4 simulates a GPP anomaly smaller than 0.5 μmol m^{-2} s^{-1}. After this, we binned the NIRv anomalies to 50 bins, removing bins with less than 2 data points. We then correlated the mean NIRv of each bin to the mean GPP of each bin.

D.2 SiB driver data and fluxes at selected EC sites.

We further assessed the SiB4 response by comparing its output to EC measurements at available European forest sites (Table S8). In these comparisons SiB4 is driven by 0.5 by 0.5 degree meteorology from ERA5, and not by the locally observed meteorology at the site, which typically reduces model skill. We nevertheless assess the original gridded SiB4 version here, since we use the model as a tool to look at integral carbon cycle impacts across Europe, and not as a local ecosystem simulator.

We summarised anomalies of SiB4 output at the EC sites, to compare them with the measured anomalies (main Table 1). The driver data anomalies are shown in Table S4 and the flux anomalies, as well as the VPD anomaly and SPEI, are shown in Table S5.

A comparison between the SiB4 anomalies and the EC anomalies shows that SiB4 correctly identifies the years and seasons with extreme events observed in the EC measurements of GPP (see Fig. S5), capturing the sign of summer and autumn NEE anomalies. The slope of the linear regression between latent heat flux anomalies and GPP anomalies at those sites are similar in SiB4 and at measurement towers as well (0.08 and 0.065 μmol m^{-1} s^{-1} / W m^{-2} for EC and SiB4, respectively), indicating a

Table S5. Similar to Table 1, but for the high-resolution SiB4, selected at the gridcells the stations are in.

	GPP (μmol m^{-2} s^{-1})		NEE (μmol m^{-2} s^{-1})		TER (μmol m^{-2} s^{-1})		SPEI (-)		VPD (hPa)	
	JJA	SON	JJA	SON	JJA	SON	JJA	SON	JJA	SON
station.name										
FR-Hes	0.49	4.07	-0.39	-2.02	0.88	6.09	-0.08	0.24	3.24	2.85
FR-Pue	-0.62	2.92	0.67	-1.73	-1.29	4.65	-0.62	0.56	5.47	1.93
IT-SR2	-2.27	2.70	1.32	-1.33	-3.59	4.03	-1.27	-0.36	4.13	1.95
BE-Vie	1.29	1.43	-0.78	-0.87	2.08	2.30	-0.22	-0.12	2.48	1.04
DE-HoH	-1.36	1.45	0.81	-1.06	-2.17	2.51	-1.16	-0.34	2.21	1.07
DE-Tha	0.38	1.93	-0.09	-1.42	0.47	3.35	-0.06	0.49	1.60	0.67
FR-FBn	-0.86	2.22	0.61	-1.23	-1.46	3.44	-0.56	0.68	4.03	1.65
SE-Htm	0.21	2.09	0.05	-1.53	0.16	3.62	-0.70	-0.49	1.37	1.03
BE-Bra	0.44	-	-0.06	-	0.50	-	-0.13	-	0.49	-
FI-Hyy	0.90	-0.05	-0.51	0.05	1.41	-0.10	-0.08	-0.24	1.58	0.08
FR-Bil	-1.90	0.63	1.38	-0.12	-3.28	0.75	-1.01	-0.89	8.37	3.52
CH-Dav	0.74	2.45	-0.37	-1.65	1.11	4.11	-0.64	-0.51	1.57	1.24
FR-Fon	-0.83	2.94	0.32	-1.67	-1.15	4.61	-0.29	-0.19	4.11	2.33
DK-Sor	0.50	2.19	-0.34	-1.43	0.84	3.62	0.06	-0.30	0.89	1.12

good water-use efficiency according to SiB4. For the 2022 drought, SiB4 tends to underestimate the SPEI levels derived at site level, suggesting that its coarser driver data have dampened the intensity of local impacts recorded in the EC-data, with likely impact on GPP as well.

Although relative GPP reductions in SiB4 are captured well, as described above, absolute GPP reductions due to drought are indeed better represented in 2018 than in 2022 (see Figure S6). We attribute this to a poorer representation of meteorological drivers, with SPEI calculated from measured data at the EC tower being structurally lower than SPEI calculated from SiB4 driver data. Partly due to this, SiB4 cannot replicate the strong decrease in GPP in the period of the strongest drought such as at Fr-Hes (Fig. S6d) . However, this does not mean that SiB4 has a poor drought response in GPP. Fig. S6 shows the SPEI anomaly of model and EC observations regressed against the corresponding GPP-anomalies, with regression coefficients given in Table S6. It shows that SiB4 captures the slope of ΔGPP/ΔSPEI well (SiB4: slope 2.0 vs EC: slope 2.5) and thus the drought response across the SPEI gradient. This, together with its high correlation with NIRV anomalies spatially, lends credence to its drought response at the larger scales we target. Note that because of the offset in the fitted ΔGPP, and the lack of "shutdown" at extreme SPEI's, we view the SiB4 GPP integrals as a lower limit to the estimates in the main text.

Table S6. Linear regression statistics of SPEI vs ΔGPP corresponding to Figure S6c

	Slope	Intercept	R	P
EC	2.5	-0.46	0.53	0.049
SiB	2.0	0.77	0.81	5E-4

Table S7. Linear regression statistics for SiB4 versus EC-measured GPP and NEE [μmol/m^2/s]

	Slope	Intercept	R	P
GPP 2018	0.56	0.32	0.86	7E-5
NEE 2018	0.51	0.20	0.81	4E-4
GPP 2022	0.24	0.26	0.52	0.057
NEE 2022	0.23	0.09	0.57	0.032

D.3 Limiting stress factors

SiB4 calculates the limitation on GPP from stress due to atmospheric water demand (VPD stress), rootzone soil moisture stress (SM stress) and temperature stress (T stress)[9]. To assess the relative importance of each of these stresses, we calculate the

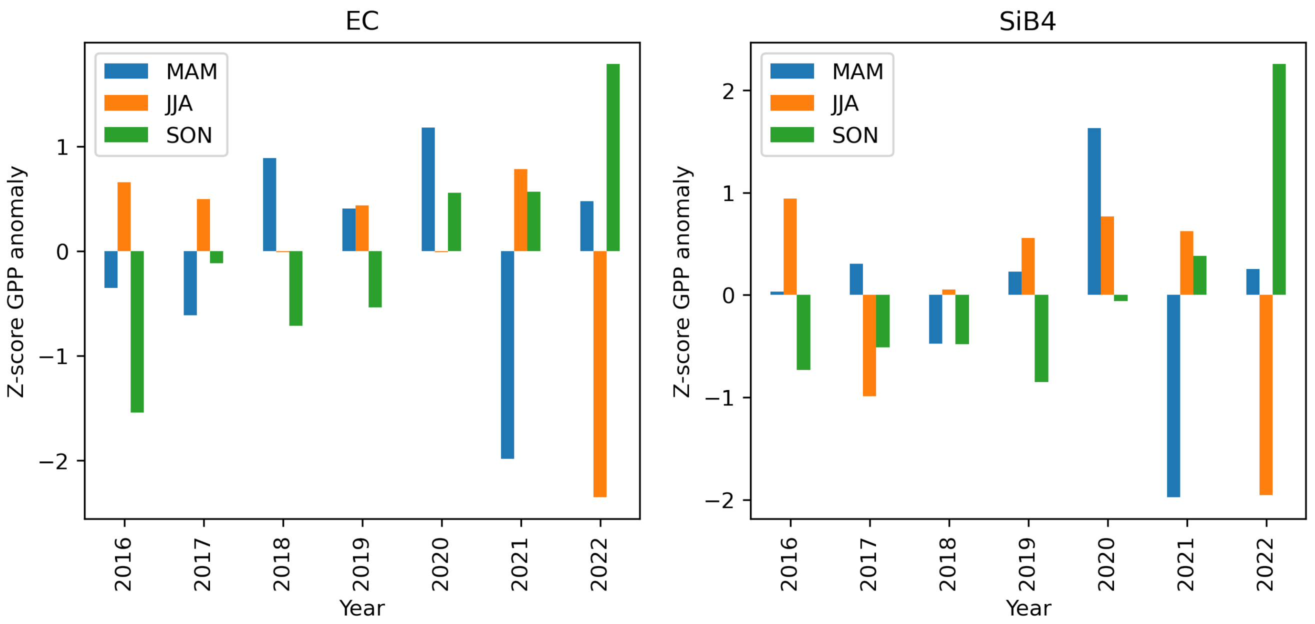

Figure S5. Z-scores (anomaly divided by the standard deviation) for GPP at EC towers and as calculated by SiB4 in different seasons over 2016-2022.

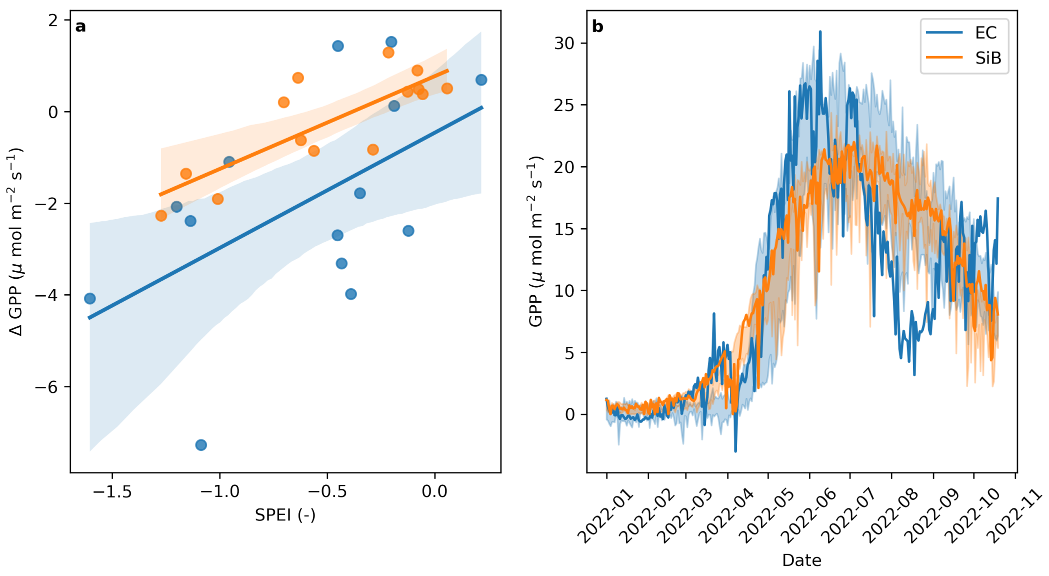

Figure S6. a) GPP anomalies in JJA 2022 as function of SPEI for EC (blue) and SiB4 (orange); b) Seasonal cycle of the site FR-Hes. The solid blue (orange) line shows the measured (simulated) GPP in 2022 and the blue (orange) shading the climatology (2016-2021, mean $\pm$ 1σ). We note that the SPEI based on site-measured meteorology is -1.7 and according to SiB4 driver data -0.16 in July of 2022.

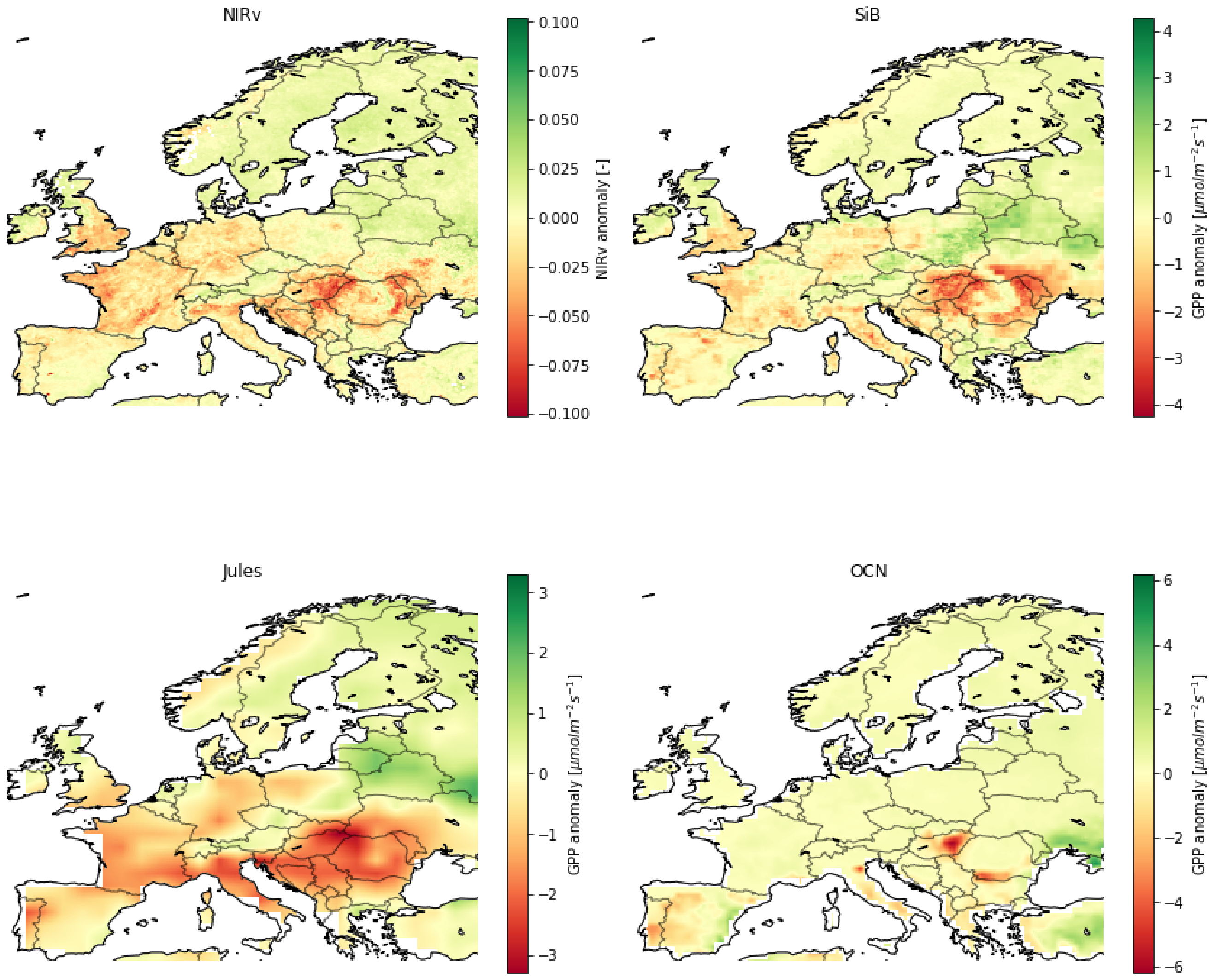

Figure S7. Anomalies in JJA 2022 compared to 2016-2021 for NIRv and GPP as simulated by the biosphere models SiB4, JULES and OCN. Negative anomalies signify reduced GPP.

relative amount of days for which each of the three stresses is limiting GPP. Figure 5 shows the result of this analysis for July and August 2018 and 2022, to assess the difference in dominant stress factor in the different years.

We note that the VPD stress denotes only the leaf evaporative demand, without any temperature dependence, even though temperature influences VPD. Additionally, We note that SiB4 does not simulate the temperature stress to be dominant, even in the southern part of Europe. This is mainly due to the calculation of stress in SiB4, as GPP is limited by the most limiting stress. Although temperature stress is present, the water-related stresses (VPD and SM stress) are more important.

D.4 Comparison to other biosphere models

We compared the SiB4 results to the biosphere models OCN[11] and JULES[12], which provide monthly mean data in near real-time. We find that OCN does not show the drought response (in GPP) that is seen in NIRv. In contrast, both SiB4 and JULES have a spatial Pearson correlation coefficient of roughly 0.4 when comparing GPP anomalies to NIRv anomalies for July 2018 and 2022. Note here that SiB4 has a nearly 120 times higher spatial resolution (0.1°x0.2° for SiB4, 1.875°x 1.25° for JULES). JULES and SiB4 show reduced GPP compared to 2019-2021 in all drought-affected areas, and higher GPP in the Northern area in the summer of 2022 (Figure S7).

Although the 2022 summer GPP response in JULES and SiB4 is similar, they show an opposite response of NEE in the Centre and Northern region, when averaged over the entire region. SiB4 estimates enhanced net uptake, compared to 2019-2021 in the North and reduced net uptake in the Centre region. This is vice-versa for JULES (Figure S8). We note that with SiB4, we show good correspondence with atmospheric CO_2 mole fraction anomalies after transport (see also Section E).

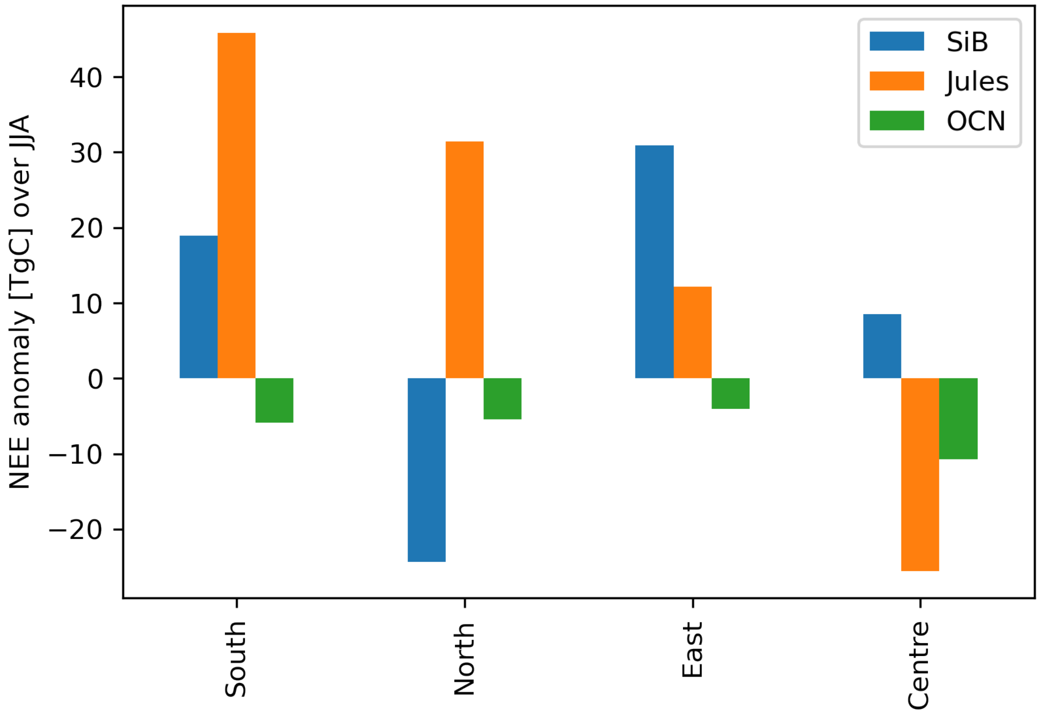

Figure S8. Total integrated 2022 summertime NEE anomalies in the different regions relative to 2016-2021 as calculated by the biosphere models SiB4, Jules and OCN. Negative numbers signify reduced carbon uptake by the biosphere.

The OCN and JULES biosphere models do not match the GPP anomalies in the 2022 warm autumn in some regions over Europe, when compared to the observed NIRv (Figure S9). Where NIRv indicates enhanced GPP over Scandinavia and central Europe (covering France, Germany through Ukraine), Jules simulates a reduced GPP over Scandinavia, SiB4 shows a less strong response, and OCN simulates a reduced GPP over the Eastern part of Europe. Notably, both the GPP response of SiB4 and Jules are similar to the observed NIRv in central Europe.

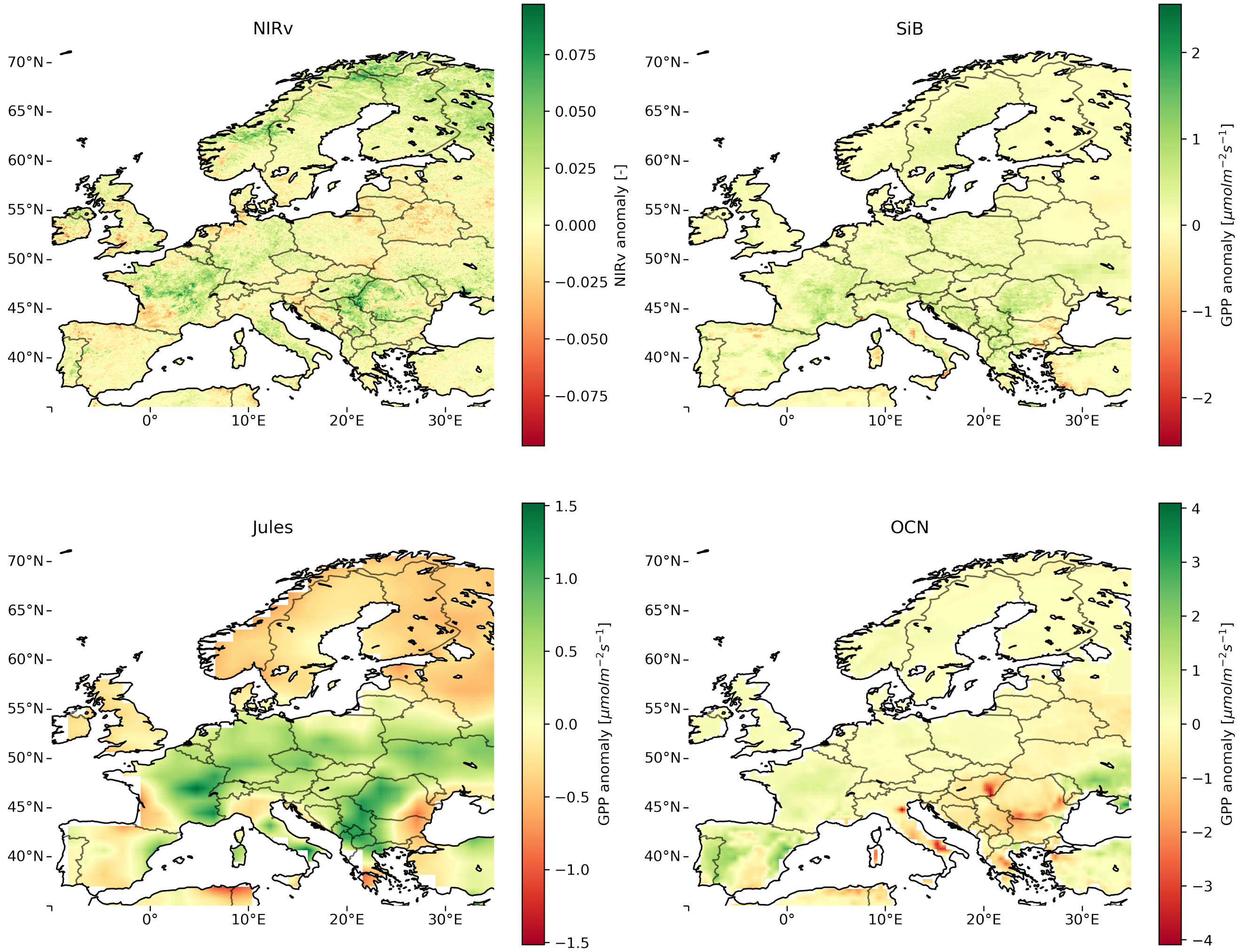

Figure S9. Anomalies in October 2022 compared to 2016-2021 for NIRv and GPP as simulated by the biosphere models SiB4, JULES and OCN. Positive anomalies signify higher than average GPP/NIRv.

E Estimates of the NEE anomaly from atmospheric measurements

Using the atmospheric CO_2 mole fraction anomalies described in C, we estimate the total biosphere flux anomaly using: 1) a box model and 2) a simple inversion method, which we describe next.

E.1 Box model

We have first calculated the biosphere CO_2 uptake anomalies using a simple box model, assuming perfect mixing in the atmospheric boundary layer. This simple model directly translates observed CO_2 mole fraction anomalies in the atmosphere to biosphere flux anomalies, and can therefore shed light on the magnitude of the biosphere flux anomalies. This is done using Equation 1.

$$\Delta CO_2 = \Delta\phi \cdot A \cdot \alpha \tag{1}$$

where ΔCO_2 is the area averaged anomaly in atmospheric CO_2 mole fraction observations at the stations [ppm/month], ϕ is the area averaged CO_2 flux [mol/m^2/month] and A represents the area [m^2] affected. α [ppm mol^{-1}] is a conversion factor for moles of carbon to ppm of CO_2 for a given volume of dry air. We use boxes the size of the four regions, with a mixing layer height of 1000m, which is very similar to the mixing layer height used in STILT (see C.1). We assume that all stations that lie within a region are equally representative of the fluxes only within that region, which is a coarse assumption. We average the observed CO_2 mole fraction anomalies per region to use in the box model. As there are no atmospheric CO_2 mole fraction measurements over the East region, we cannot use the box model to estimate the biosphere anomaly over this region. To quantify the impact over the drought-affected area, we use the simulated anomaly by SiB4 in this region in the box model.

E.2 Simple inversion

We give a second estimate of the European NEE anomaly informed by atmospheric CO_2 mole fraction measurements using a simple inversion, in which the mismatch between between the simulated and observed CO_2 mole fraction anomalies is minimised. We do this by scaling the NEE anomalies as simulated by SiB4 (See D) in each individual region to be optimally consistent with the observed atmospheric mole fraction anomalies. This optimisation is done using a Kalman filter[13], which minimises Equation 2:

$$J(x) = \left(y^0 - \mathscr{H}(x)\right)^T R^{-1}\left(y^0 - \mathscr{H}(x)\right) + (x - x^b)^T P^{-1}(x - x^b) \tag{2}$$

where y^0 are the atmospheric CO_2 mole fraction anomalies (see Section C), with a covariance R. We set R to a diagonal matrix with an uncertainty of 1.5 ppm, which is similar to Munassar et al (2023)[14]. x is a vector of four scaling factors per month (one per region) of the SiB4 NEE anomalies (relative to 2019-2021). $\mathscr{H}$ denotes the observation operator, translating the state vector x to atmospheric mole fraction anomalies. We calculated $\mathbf{H}$, the linearised matrix representation of $\mathscr{H}$, by transporting the NEE anomalies from SiB4 to all stations using STILT, driven by IFS. x^b is the prior estimate of the state vector, which we set to 0 (no anomaly in NEE), meaning that the calculated anomaly is driven only by atmospheric measurements and SiB4 patterns, but not SiB4 magnitude. x^b has a covariance P, which we set to 1.0. This large covariance indicates that little weight is given to the prior estimate of 0 anomaly, resulting in a estimate that is mainly constrained by atmospheric observations. To limit the influence of large biases in observed CO_2 due to fossil fuel plumes, as well as to minimise misrepresented atmospheric transport, we remove observations that have a deviation from the first-guess (SiB4) of more than 3σ. Note that doing a full-fledged inversion, such as in Smith et al (2020, referred to as SM2020 from hereon)[10], requires detailed (non-climatological) background CO_2 fields, anthropogenic and oceanic CO_2 fluxes.

E.3 Comparison to other inverse estimates of the drought of 2018

We validate our quick inversion with a more complex inversion, as described by SM2020, who use a global atmospheric transport model, as well as influence by fluxes outside our study domain. Moreover, they included anthropogenic and oceanic fluxes and used a lagged ensemble Kalman filter[13]. Their transport was done by TM5[15] at 1x1 degree resolution, whereas we use STILT at 1/12 latitude by 1/8 degree longitude.

Notwithstanding these differences in methodology, we find a very similar response of the European NEE over the drought-affected area in 2018 (see Section A). This is shown in Figure S10. The figure also shows the anomaly as calculated by SiB4, which shows a remarkable resemblance to both inverse results. Note that the quick inversion starts from a 0 anomaly, and the sign and magnitude are both only informed by atmospheric measurements. The fact that SiB4 and the quick inversion show such resemblance in JJA improves our faith in the ability of SiB4 to simulate NEE anomalies.

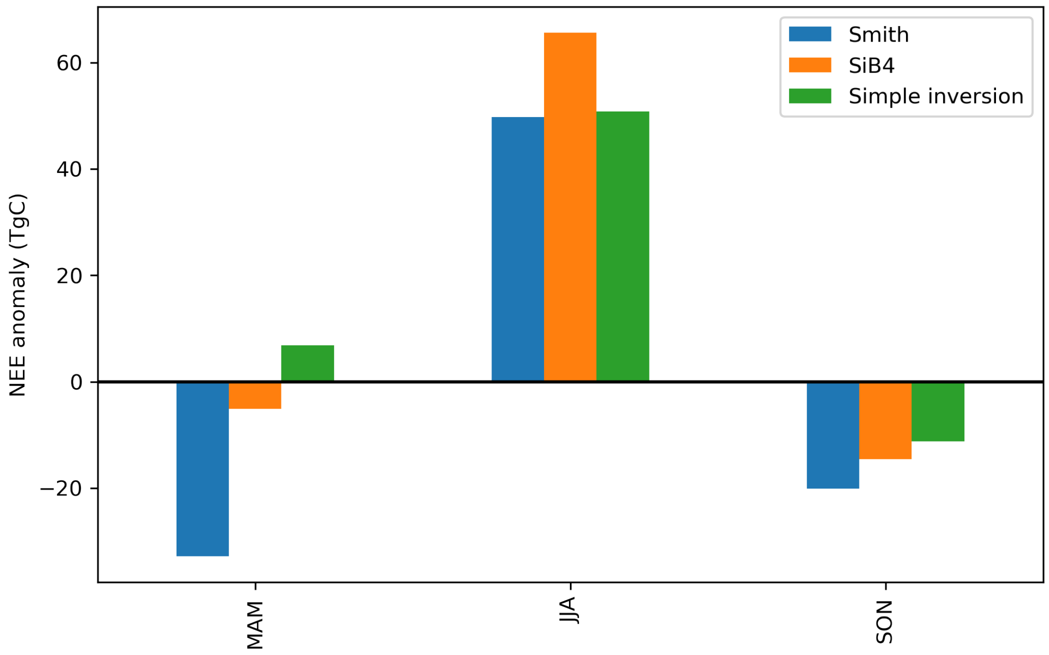

Figure S10. NEE anomalies for the drought-affected area in 2018 (North and Centre) according to the inversion by SM2020, according to SiB4 and according to the quick inversion described here (Simple inversion). The anomalies are relative to 2019-2021 for SiB and Inv, but relative to 2012-2017 for SM2020.

F Eddy Covariance data

Eddy-covariance data for 14 forest sites (Table S8) in Europe was used. Most sites experienced decreased precipitation, enhanced incoming radiation and higher air temperatures in summer and autumn 2022, compared to 2016-2021 (excluding 2018). This is shown in Table S9. For all analysis, only day-time data (incoming solar radiation > 10 $Wm^{-2}s^{-1}$) is used.

Due to the high temperatures in autumn 2022, most of the EC sites experienced higher GPP, but also respiration, compared to the climatology. This is shown in Figure S11.

To calculate SPEI from the EC data, the R SPEI package was used (`https://cran.r-project.org/web/packages/SPEI/SPEI.pdf`, with potential evapotranspiration calculated using the Hargreaves method. For consistency, the SPEI for the SiB4 simulations at the EC sites was done following the same processing pipeline. For all analysis, we use 3-month SPEI.

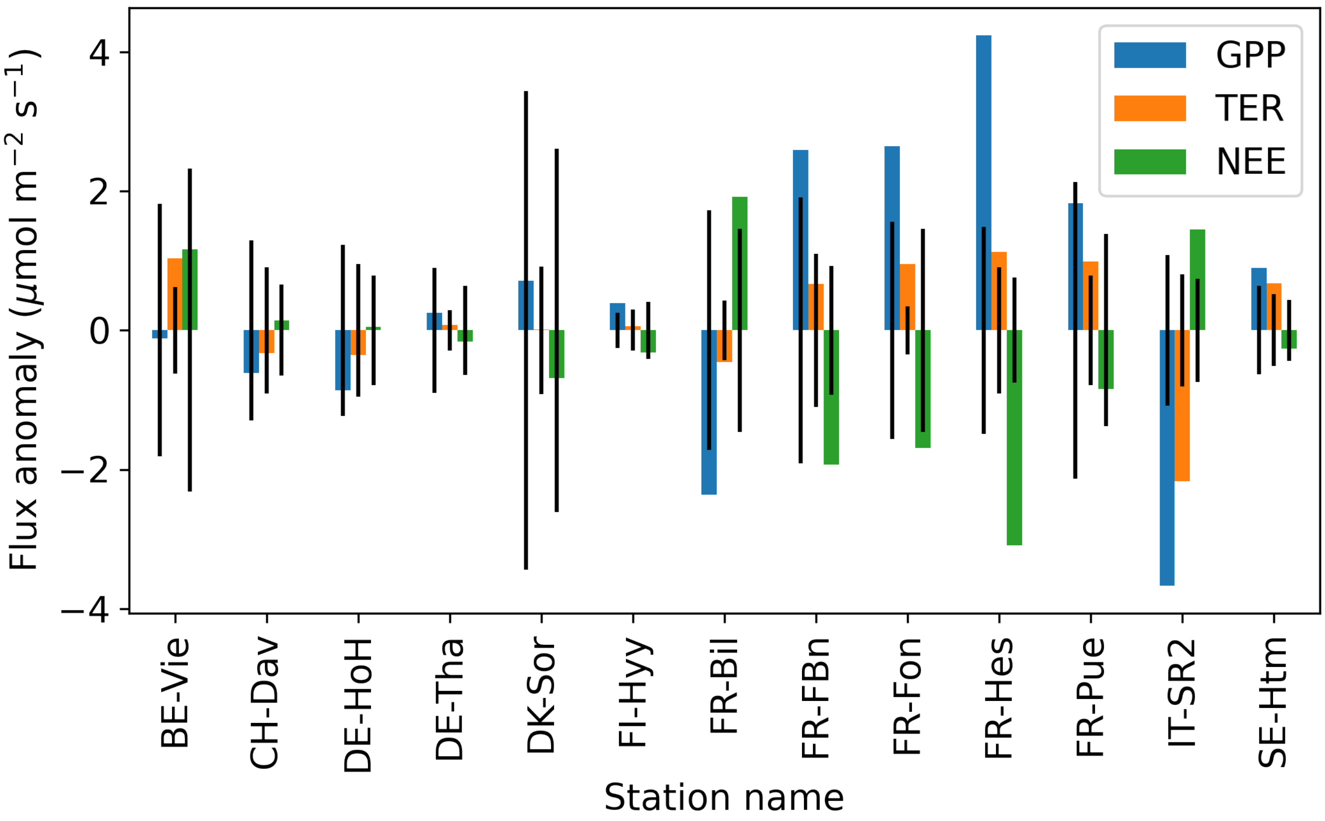

Figure S11. GPP, TER and NEE anomalies in autumn (SON) 2022, compared to 2019-2021. The black error bars indicate the standard deviation of the climatology.

Table S8. Used eddy covariance (EC) sites and their location in latitude (Lat, degrees north), longitude (Lon, degrees east), altitude above ground level (alt), as well as plant functional type (PFT). *EBF: Evergreen Broadleaf forest

Station long Name	Short Name	Lat (degree north)	Lon (degree east)	alt (m.a.g.l.)	pft
De Inslag Brasschaat	BE-Bra	51.31	4.52	20	Coniferous forest
Vielsalm	BE-Vie	50.3	56.0	490	Mixed Forest
Hohes Holz	DE-HoH	52.09	11.22	239	Deciduous forest
Tharandt	DE-Tha	50.96	13.57	385	Coniferous forest
Soroe	DK-Sor	55.49	11.64	40	Deciduous forest
Hyytiälä	FI-Hyy	61.85	24.29	181	Boreal pine forest
Salles	FR-Bil	44.49	-0.96	38	Coniferous forest
Barbeau	FR-Fon	48.48	2.78	92	Deciduous forest
Hesse	FR-Hes	48.67	7.06	300	Deciduous forest
San Rossore 2	IT-SR2	43.73	10.29	4	Coniferous forest
Hyltemossa	SE-Htm	56.10	13.42	104	coniferous forest
Davos	CH-Dav	46.82	9.86	1639	Coniferous forest
Puéchabon	FR-Pue	43.74	3.60	270	Evergreen broadleaf forest
Fontblanche	FR-Fbn	43.24	5.68	420	Coniferous forest + EBF*

G Near-infrared reflectance of vegetation

The near infrared reflectance of vegetation (NIRv) is shown to be a good proxy for leaf phenology, and thereby for GPP[16]. NIRv can give information on the seasonal variation of GPP across a range of ecosystems[17,18], but more importantly also on drought impacts[10,19]. Because of this relation, we use NIRv to assess spatio-temporal variability of GPP anomalies and the progression of the drought. Hence, we can also use NIRv as an independent validation of the SiB4 GPP response to the drought.

G.1 NIRv-GPP anomalies

From the reduction in observed NIRv, we calculate reductions in GPP over the drought-affected area. For this, we use the linear relation between NIRv and GPP, similar to SM2020. For completeness, the methodology is repeated briefly here. We first calculate the linear regression slope between NIRv and GPP at EC sites for all available site-months. We average these slopes per plant functional type (PFT), to obtain the PFT-specific NIRv-GPP relationship, allowing for an offset (GPP = slope * NIRv + offset) by not forcing this relation through the origin. From the NIRv anomalies, we then calculate the corresponding GPP anomalies, which we integrate over the four regions (see Section A).

To assess the uncertainty on this integrated estimate of GPP anomaly, we calculate the GPP anomaly using four different methods: 1) we use the slopes that we used in SM2020, 2) We used the slopes from SM2020, and linearly detrend the NIRv signal pixel-wise; 3) We used slopes calculated with EC data up to 2023 (see Table S8); 4) We used the slopes from 3) and we linearly detrended NIRv. By calculating the GPP anomaly using these four methods, we obtain an average and a standard deviation. We find that all four methods result in GPP anomalies that differ in the order of 10-20% of their magnitude and are consistent with SM2020.

Over the entire growing season (March-Nov), we find a GPP anomaly of -549.0 $\pm$ 64.2 TgC in 2022, compared to an anomaly of -369.3 $\pm$ 72.7 TgC in 2018.

A more in-depth analysis of NIRv anomalies is discussed in the following Section.

G.2 NIRv anomalies

NIRv anomalies in 2022, relative to 2001-2021, show significant reductions in NIRv in the Centre, South and Eastern regions (see Figure S13). The figure shows the enhanced uptake in spring 2018 in the Northern region, as well as the reductions in summertime of that year. We find reductions in NIRv in the Centre region that are smaller in 2022 than in 2018, but both years show reductions outside the 2σ range.

Contrary to the enhanced uptake in spring 2018 in the Northern region, NIRv retrievals indicate reduced uptake from April onwards, especially in the Eastern and Southern region. Peak reductions in NIRv are observed in July and August in the Centre, East and Southern region, after which a prolonged growing season, indicated by positive NIRv anomalies, starts in from October 2022. Assessing the anomalies per land-use type in the CORINE database[20], this positive anomaly is especially prominent for broadleaved forests, C3 grasses and C3 crops, with positive anomalies of $\pm$ 2σ for each of these land-use types in November over the drought-affected area (see also Figure S13). Contrary, evergreen forests and C4 crops show only small recovery, with anomalies of roughly 1σ.

Table S9. Precipitation (P), Incoming solar radiation (Rg) and air temperature (Tair) anomalies during summer (JJA) and autumn (SON) 2022, relative to 2016-2021, excluding 2018, as measured at the used EC towers. Note that for precipitation, also nighttime data is included. The precipitation anomaly is calculated as (P_{2022} - P_{clim}) / P_{clim}, where P is the average precipitation over the selected months and years (indicated by the subscript)

	P		Rg (W/m^2)		Tair (K)	
	JJA	SON	JJA	SON	JJA	SON
station.name						
BE-Vie	-0.61	-0.29	51.83	-0.62	1.70	1.50
FR-FBn	-0.44	-0.50	5.90	2.95	2.07	1.42
BE-Bra	-0.59	-	42.51	-	0.43	-
IT-SR2	-0.66	-0.61	15.58	15.43	2.07	2.12
FR-Fon	-0.49	-0.17	52.72	27.57	2.09	2.30
FR-Bil	-0.54	-0.08	17.67	-19.97	3.79	2.53
FI-Hyy	-0.22	-0.12	36.24	9.14	1.27	0.42
DE-Tha	-0.05	1.45	-14.21	-13.82	0.80	1.40
FR-Hes	-0.88	0.42	57.96	57.69	1.53	2.98
DE-HoH	-0.60	0.43	39.54	35.96	1.99	1.57
FR-Pue	-0.75	-0.56	23.83	-32.14	3.43	2.18
CH-Dav	-0.54	-0.43	56.88	11.68	1.87	2.29
SE-Htm	-0.51	-0.14	35.35	38.78	0.71	1.25
DK-Sor	-0.58	-0.49	27.28	38.47	0.65	1.52

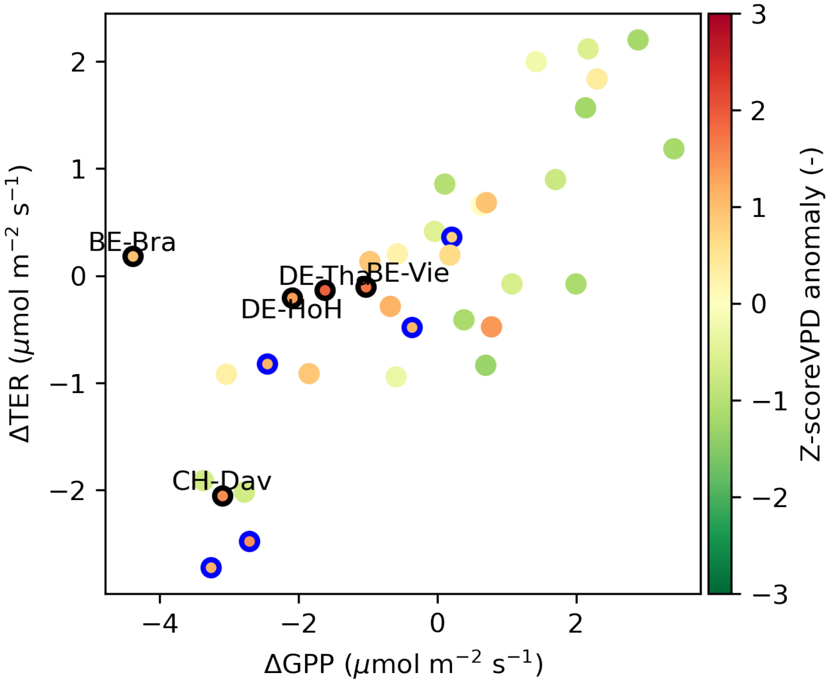

Figure S12. GPP and TER anomalies with respect to 2019-2021 for EC sites that fall within the Centre region. Anomalies in 2018 are indicated by blue edgecolours and 2022 by black edgecolours. For 2022 the individual sites are labeled. the Z-score (anomaly divided by the standard deviation) of VPD anomalies are shown as colours.

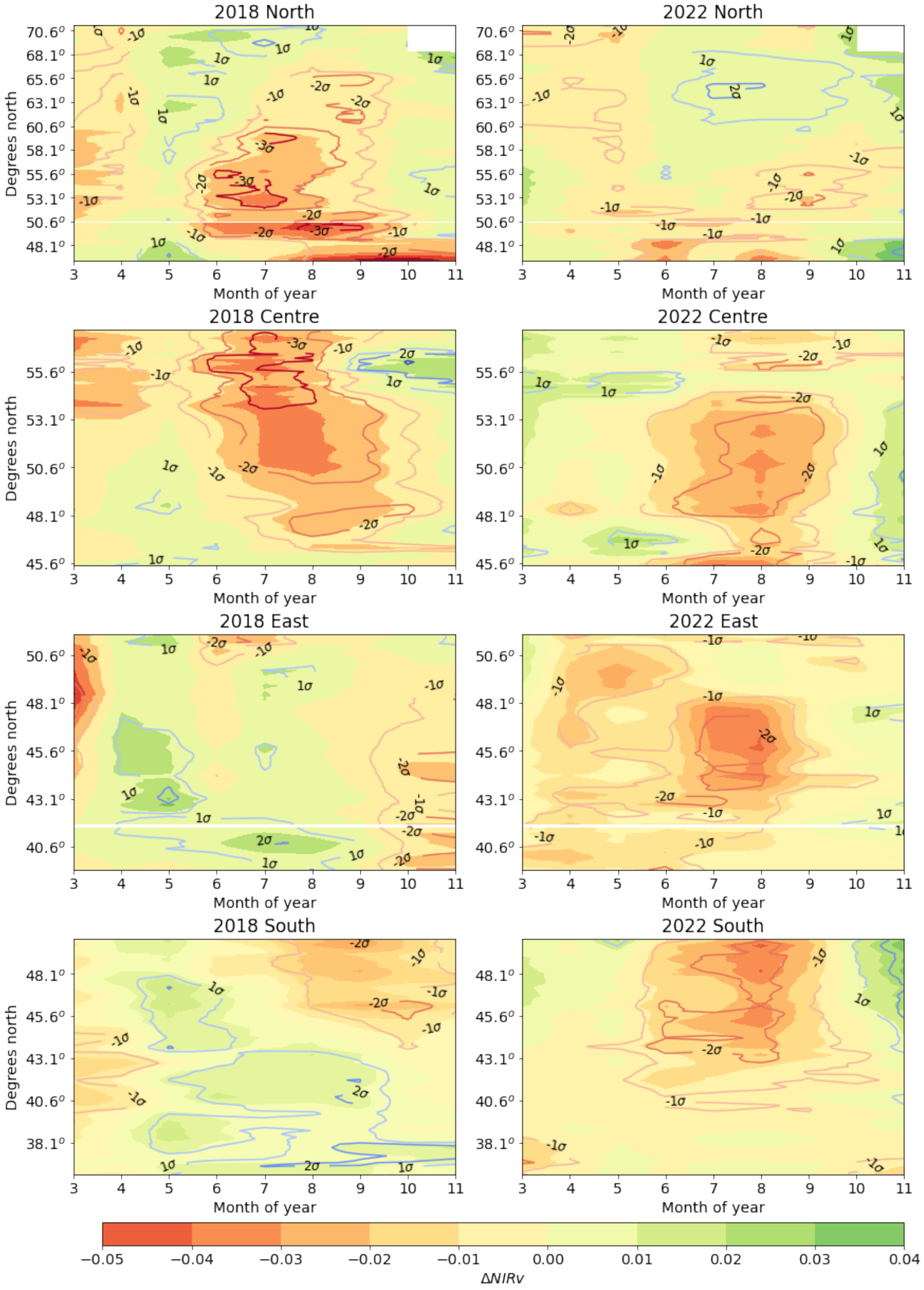

Figure S13. Detrended NIRv anomalies over the drought regions, taken as zonal means ('Hovmöller diagram'). The contourlines indicate the Z-scores (anomaly divided by the standard deviation).

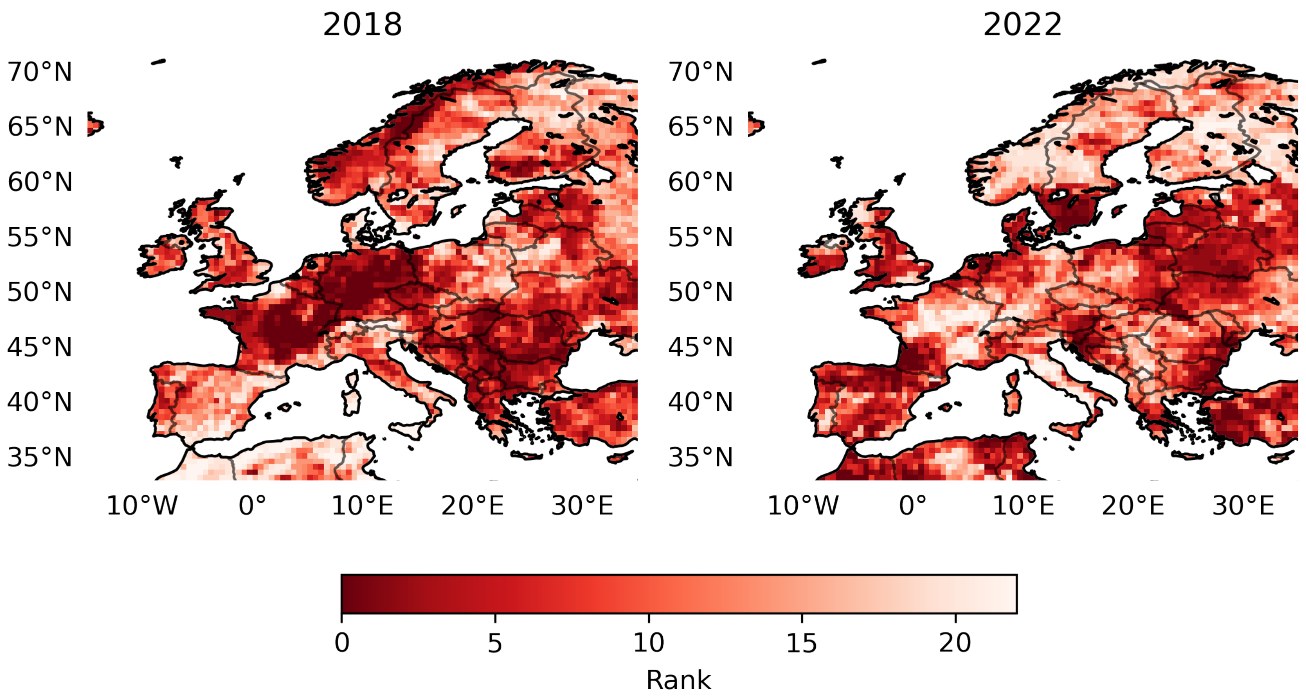

Figure S14. Ranked NIRv anomalies with respect to 2001-2022 for JJA 2018 and 2022, with lower values indicating lower ranked NIRv observed over that respective gridcell.

Due to their reduced NIRv, both the summer of 2018 and 2022 ranked amongst the lowest of the past two decades in NIRv in their respective drought-affected areas. This is shown in Figure S14, indicating the relative severity of both droughts on carbon uptake over Europe. Contrary, autumn has the highest NIRv values, indicating delayed leaf senescence S15.

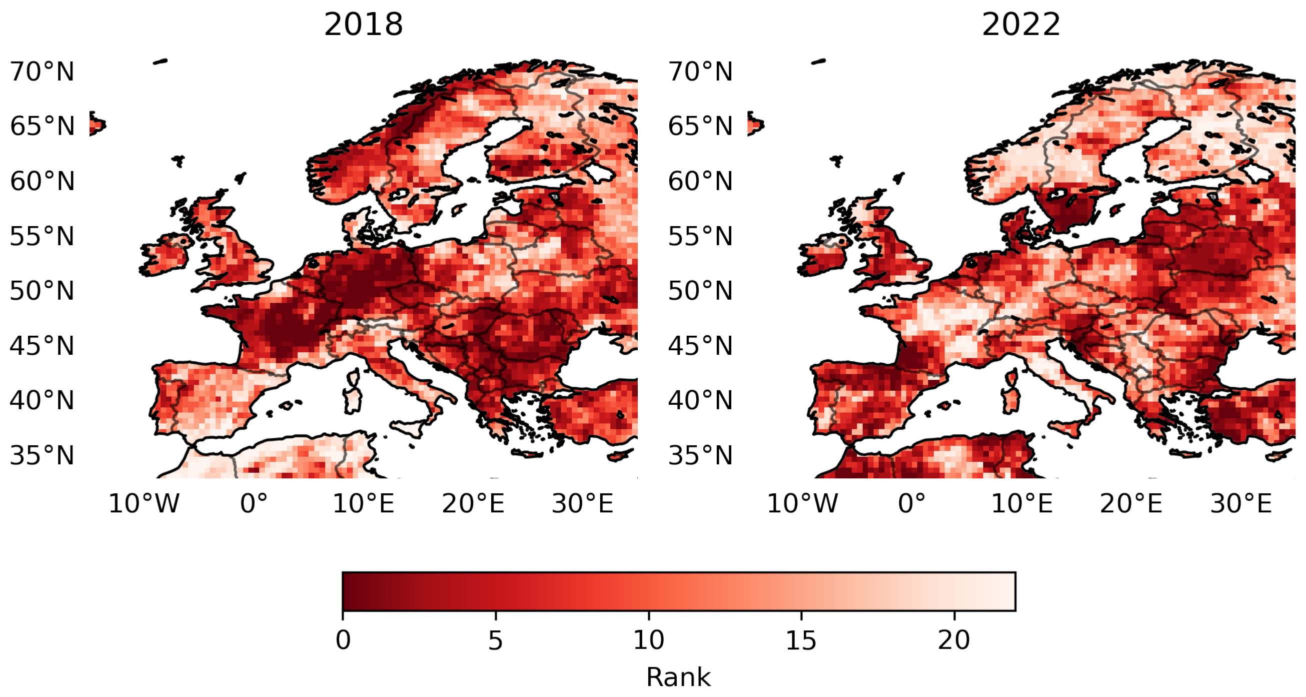

Figure S15. Same as Figure S14 but for SON.

Table S10. Estimated anomalies over JJA 2022 from NIRv (GPP), SiB4 (NEE) and the simple inversion (NEE) in TgC, relative to 2019-2021, and total emissions from wildfires (TgC), according to GFAS. Note that we do not have atmospheric mole fraction observations of CO_2 over the East region, and therefore cannot constrain it in the inversion. Negative GPP anomalies are lower than average values, positive NEE anomalies signify less net carbon uptake than average in 2019-2021.

Region	ΔGPP (TgC)	ΔNEE (TgC) (SiB4)	ΔNEE (TgC) (Inv)	Fire (TgC)
Centre	-109.4	7.8	8.1	0.23
South	-148.4	9.4	10.3	4.8
East	-195.0	38.6	-	0.20
Total	-452.9	55.8	18.4	5.2

H Carbon balance impacts

Based on the NIRv-GPP estimate (Section G), SiB4 (Section D) and the simple inversion (Section E.2), we provide a first estimate of the impact of the drought of 2022 on the European carbon budget in summer (Table S10) and autumn Table S11) in the drought-affected area.

Table S11. Same as Table S10 but for ON. Positive GPP anomalies are higher than average values, negative NEE anomalies signify more net carbon uptake than average in 2019-2021.

Region	ΔGPP (TgC)	ΔNEE (TgC) (SiB4)	ΔNEE (TgC) (Inv)	Fire (TgC)
Centre	38.7	-2.8	-2.7	0.04
South	30.8	-3.4	-3.6	0.13
East	18.0	-5.2	-	0.06
Total	87.5	-11.4	-6.3	0.23

I Fires

To analyse the fire contribution to the total CO_2 flux anomaly over Europe, we take fires from the GFAS database[21], processed following vdW2022. A first comparison between GFAS and GFED[22] shows good agreement between the two, except for over France, where an unresolved issue was found in the GFED data (Guido van der Werf, 2022, personal communication). In contrast, GFAS emissions and emissions estimated by Vallet et al. (2023)[23] are consistent in France.

Anomalously large fires in Europe during the drought of 2022 accounted to $\pm$5.2 TgC emissions, additional to the reduced uptake in the European summer of 2022. CO_2 emissions from fires mainly took place in the South region, where 4.8 TgC was emitted (see also S10. The location of the fires is shown in Figure S16. The figure shows intense fires in the South of France and the Northeast of the Iberian peninsula.

Additionally, we use fire counts from the Visible Infrared Imaging Radiometer Suite[25]. Cumulative fire counts for Europe are shown in Figure S18.

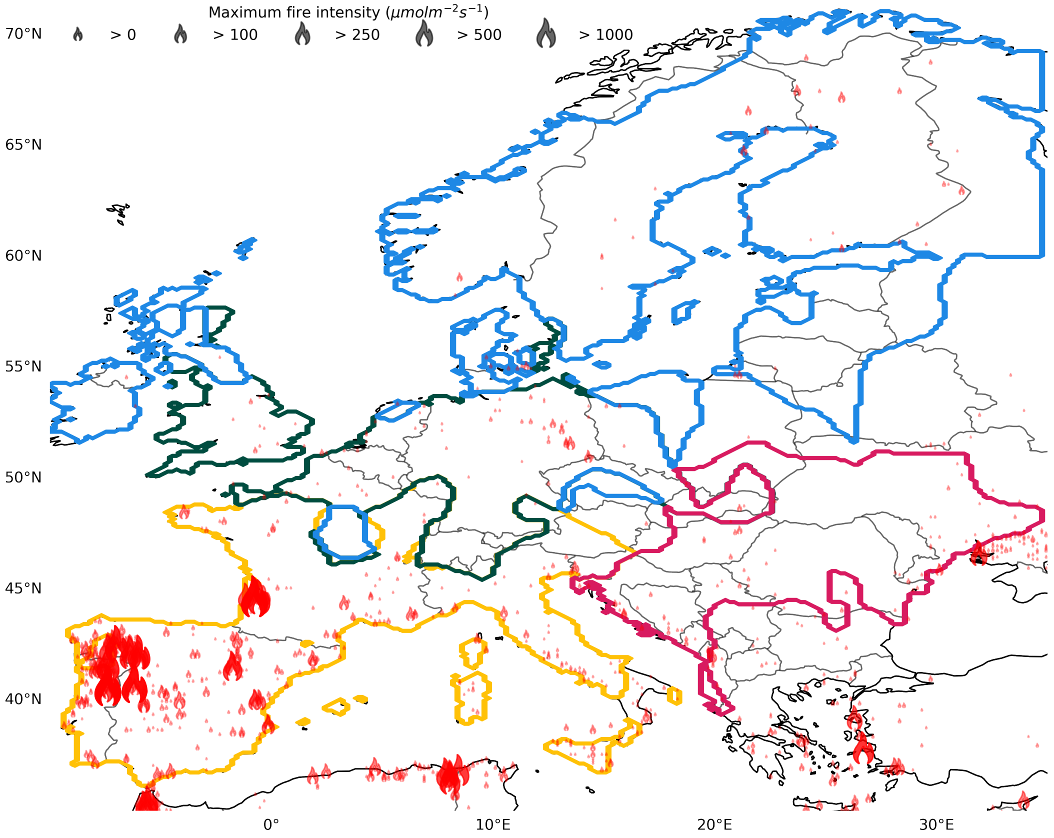

Figure S16. Location and intensity of summer (JJA) 2022 wildfires. The contours indicate the different regions; with the North coloured blue, the centre green, the South yellow and the East coloured red. The size of the marker indicate the maximum intensity, measured as μmol C m^{-2} s^{-1}

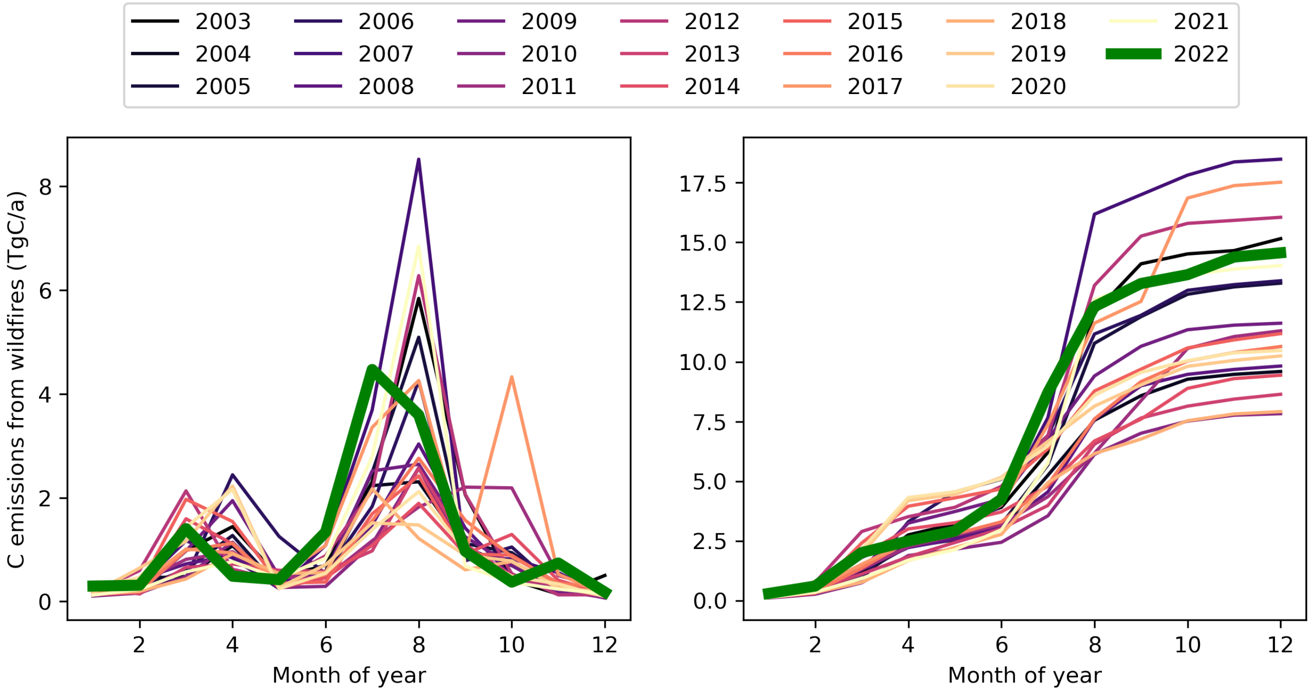

Figure S17. Monthly (left) and cumulative (right) total yearly C emissions due to fires over Europe from GFAS[7,24].

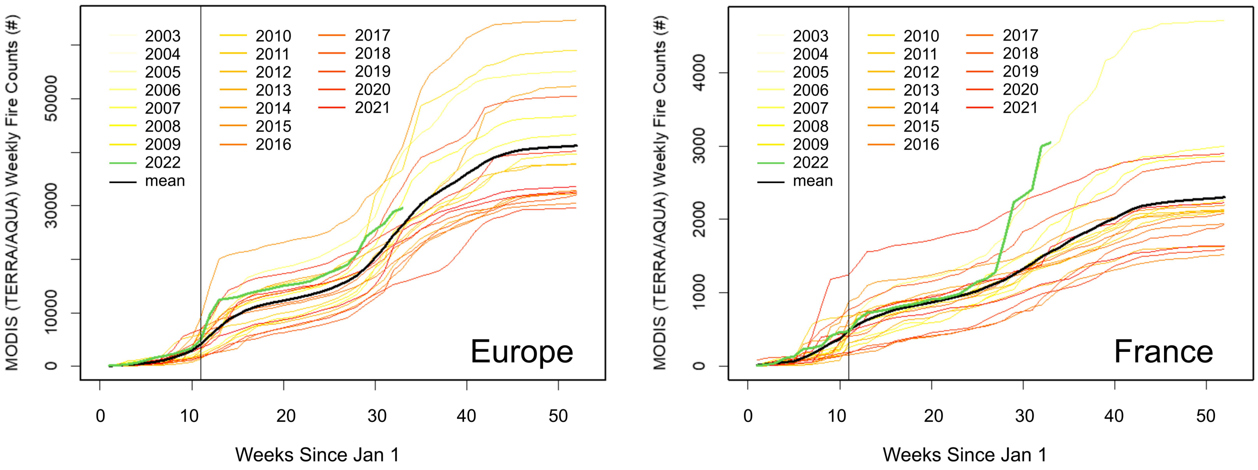

Figure S18. Cumulative fire counts from VIIRS over Europe (left) and France (right).

J Color blind-friendly main figures

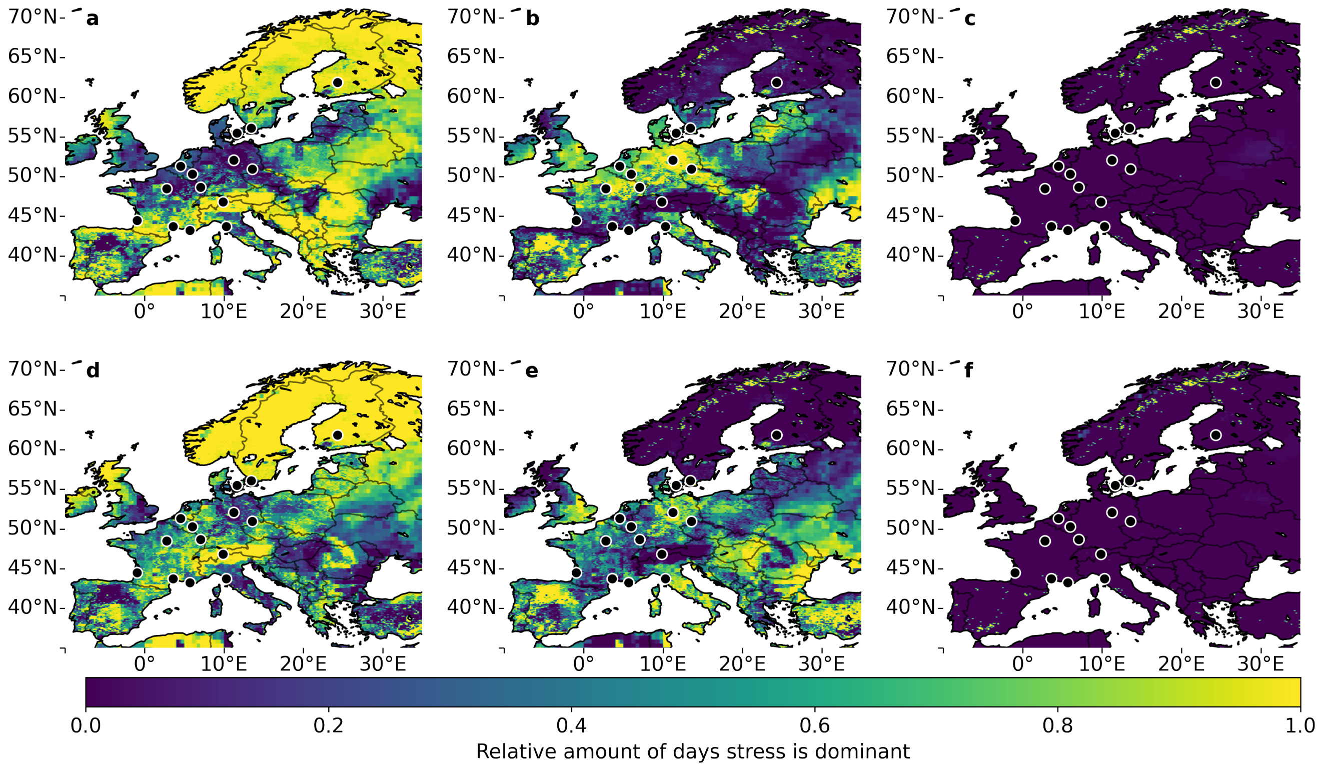

Figure S19. Figure 5 a and b of the main text in a colorblind friendly colormap. The figures show relative amount of days the stress from VPD, Soil moisture and Temperature is limiting in 2018 (a, b, c) and 2022 (d, e, f), respectively. Lower values indicate more limiting stress.

Supplementary References

1. Avitabile, V. *et al.* Comparative analysis and fusion for improved global biomass mapping (2014).
2. Entekhabi, D., Njoku, E. & O'Neill, P. The Soil Moisture Active and Passive Mission (SMAP): Science and applications. In *2009 IEEE Radar Conference*, vol. 674, 1–3, DOI: 10.1109/RADAR.2009.4977030 (IEEE, 2009).
3. Muñoz-Sabater, J. *et al.* ERA5-Land: a state-of-the-art global reanalysis dataset for land applications. *Earth Syst. Sci. Data* **13**, 4349–4383, DOI: 10.5194/essd-13-4349-2021 (2021).
4. Hersbach, H. *et al.* The ERA5 global reanalysis. *Q. J. Royal Meteorol. Soc.* **146**, 1999–2049, DOI: 10.1002/qj.3803 (2020).
5. Munassar, S. *et al.* Net ecosystem exchange (NEE) estimates 2006–2019 over Europe from a pre-operational ensemble-inversion system. *Atmospheric Chem. Phys.* **22**, 7875–7892, DOI: 10.5194/acp-22-7875-2022 (2022).
6. Lin, J. C. *et al.* A near-field tool for simulating the upstream influence of atmospheric observations: The Stochastic Time-Inverted Lagrangian Transport (STILT) model. *J. Geophys. Res. Atmospheres* **108**, 4493, DOI: 10.1029/2002jd003161 (2003).
7. van der Woude, A. M. *et al.* Near real-time CO_2 fluxes from CarbonTracker Europe for high resolution atmospheric modeling. *Earth Syst. Sci. Data Discuss.* **2022**, 1–38, DOI: 10.5194/ESSD-15-579-2023 (2022).
8. Friedlingstein, P. *et al.* Global Carbon Budget 2021. *Earth Syst. Sci. Data* **14**, 1917–2005, DOI: 10.5194/essd-14-1917-2022 (2022).
9. Haynes, K., Baker, I. & Denning, A. S. The Simple Biosphere Model, Version 4.2: SiB4 Technical description. *Colo. State Univ.* (2020).
10. Smith, N. E. *et al.* Spring enhancement and summer reduction in carbon uptake during the 2018 drought in northwestern Europe. *Philos. Transactions Royal Soc. B* **375**, 20190509, DOI: 10.1098/rstb.2019.0509 (2020).
11. Zaehle, S. & Friend, A. D. Carbon and nitrogen cycle dynamics in the O-CN land surface model: 1. Model description, site-scale evaluation, and sensitivity to parameter estimates. *Glob. Biogeochem. Cycles* **24**, n/a–n/a, DOI: 10.1029/2009GB003521 (2010).
12. Clark, D. B. *et al.* The Joint UK Land Environment Simulator (JULES), model description – Part 2: Carbon fluxes and vegetation dynamics. *Geosci. Model. Dev.* **4**, 701–722, DOI: 10.5194/gmd-4-701-2011 (2011).
13. Peters, W. *et al.* An ensemble data assimilation system to estimate CO 2 surface fluxes from atmospheric trace gas observations. *J. Geophys. Res.* **110**, D24304, DOI: 10.1029/2005JD006157 (2005).
14. Munassar, S. *et al.* Why do inverse models disagree? A case study with two European CO 2 inversions. *Atmospheric Chem. Phys.* **23**, 2813–2828, DOI: 10.5194/acp-23-2813-2023 (2023).
15. Krol, M. *et al.* The two-way nested global chemistry-transport zoom model TM5: algorithm and applications. *Atmospheric Chem. Phys.* **5**, 417–432, DOI: 10.5194/acp-5-417-2005 (2005).
16. Badgley, G., Field, C. B. & Berry, J. A. Canopy near-infrared reflectance and terrestrial photosynthesis. *Sci. Adv.* **3**, e1602244, DOI: 10.1126/sciadv.1602244 (2017).
17. Turner, A. J. *et al.* A double peak in the seasonality of California's photosynthesis as observed from space. *Biogeosciences* **17**, 405–422, DOI: 10.5194/bg-17-405-2020 (2020).
18. Mengistu, A. G. *et al.* Sun-induced fluorescence and near-infrared reflectance of vegetation track the seasonal dynamics of gross primary production over Africa. *Biogeosciences* **18**, 2843–2857, DOI: 10.5194/bg-18-2843-2021 (2021).
19. Buitink, J. *et al.* Anatomy of the 2018 agricultural drought in the Netherlands using in situ soil moisture and satellite vegetation indices. *Hydrol. Earth Syst. Sci.* **24**, 6021–6031, DOI: 10.5194/hess-24-6021-2020 (2020).
20. Bossard, M., Feranec, J. & Otahel, J. CORINE land cover technical guide – Addendum 2000 Part I State-of-play production methods of the CORINE land cover database (2000).
21. Di Giuseppe, F., Rémy, S., Pappenberger, F. & Wetterhall, F. Using the Fire Weather Index (FWI) to improve the estimation of fire emissions from fire radiative power (FRP) observations. *Atmospheric Chem. Phys.* **18**, 5359–5370, DOI: 10.5194/acp-18-5359-2018 (2018).
22. van der Werf, G. R. *et al.* Global fire emissions estimates during 1997–2016. *Earth Syst. Sci. Data* **9**, 697–720, DOI: 10.5194/essd-9-697-2017 (2017).
23. Vallet, L. *et al.* High resolution data reveal a surge of biomass loss from temperate and Atlantic pine forests, seizing the 2022 fire season distinctiveness in France. *EGUsphere* **2023**, 1–39, DOI: 10.5194/egusphere-2023-529 (2023).

24. Di Giuseppe, F., Rémy, S., Pappenberger, F. & Wetterhall, F. Combining fire radiative power observations with the fire weather index improves the estimation of fire emissions. *Atmospheric Chem. Phys. Discuss.* 1–16, DOI: 10.5194/acp-2017-790 (2017).

25. Schroeder, W. & Giglio, L. NASA VIIRS Land Science Investigator Processing System (SIPS) Visible Infrared Imaging Radiometer Suite (VIIRS) 375 m & 750 m Active Fire Products: Product User's Guide Version 1.4 (2018).
